# The Epidemic of Ergotism in Russia

**Published:** 1881-04

**Authors:** 


The Epidemic of Ergotism in Russia.—This epidemic occurred in the autumn of 1879 in the neighborhood of Novgorod. In the district attacked, an inhospitable climate and a marshy soil were combined with poverty, dirt and general unhealthy conditions among the villagers. Of nineteen cases in which the symptoms were strongly marked, four died. In other sixteen cases the symptoms were less developed, and probably as many more escaped observation. In these slighter cases the symptoms were diarrhœa (in 70 per cent.), weakness, more especially in the hands and feet, occasional attacks of giddiness, headache, sleeplessness, and deadness of the fingers, with formication under the skin. All had, up to their seizure, eaten fresh-ground, unkilned rye, and the symptoms quickly disappeared under the use of laxatives and opiates, and the withdrawal of bread containing ergot. In the first-mentioned nineteen cases, the symptoms were severe; racking pains in the extremities, severe headache, great thirst and utter prostration, weakness of intellect and melancholia. Tonic and clonic spasms, preceded by dyspnœa, deadness in the extremities, and cold sweats attacked the flexor muscles of the limbs, the extensors being unaffected. The respirations were 14-16; maximum temperature, 99·8°; pulse slow and weak. The fatal cases, an old man and two children in one family, and a woman in another, died, three of them in a comatose condition and one during a convulsive fit. The treatment was as above, with subcutaneous injections of morphia and inhalations of chloroform, followed by tonics and improvement of hygienic conditions. The quantity of ergot presented in the rye was about 7 per cent., and two dogs fed with it each showed on the seventh week a gangrenous ulcer on one paw. On the withdrawal of the ergot bread from one dog, recovery followed in two months; while in the other, fed as before, the gangrene advanced, convulsions appeared, and death followed by way of coma in the tenth week. The *post-mortem* appearances were: brain and meninges anæmic, arteries quite empty, veins full of dark fluid blood, heart empty, lungs, liver and spleen hyperæmic, intestinal mucous membrane congested, but neither it nor the liver showing any gangrenous spots such as ha ve been described.—*London Medical Record.*

